# Hot Deformation Behavior of AA3102 Aluminum Alloy: Constitutive Modeling, Microstructural Evolution and Numerical Simulation

**DOI:** 10.3390/ma19173710

**Published:** 2026-08-31

**Authors:** Xianzheng Liu, Nashrah Hani Jamadon, Xiaoming Liu, Dongpo Wei, Chao Jin, Rongji Tang, Liancheng Zheng, Chaowei Liu, Lihua Jiang, Zhenhua Liu

**Affiliations:** 1 School of Mechanical Engineering, Shandong Huayu University of Technology, Dezhou 253034, Chinawei31028@yeah.net (D.W.);; 2 Department of Mechanical and Manufacturing Engineering, Faculty of Engineering and Built Environment, Universiti Kebangsaan Malaysia, Bangi 43600, Selangor, Malaysia; 3 School of Electrical and Electronic Engineering, Zhongyuan University of Science and Technology, Xuchang 461000, China

**Keywords:** AA3102 aluminum alloy, hot deformation, constitutive modeling, processing maps, dynamic recrystallization, finite element simulation

## Abstract

This study systematically investigates the hot deformation behavior and microstructural evolution of AA3102 aluminum alloy through isothermal uniaxial compression tests conducted at 400–550 °C and strain rates of 0.01–10 s^−1^. Previous studies on 3xxx-series Al–Mn alloys have mainly considered temperature and strain-rate effects while neglecting strain-dependent material parameter variations, limiting prediction accuracy under large deformation and obscuring dynamic softening mechanisms. Here, the experimental flow stress data were corrected for interfacial friction and adiabatic temperature rise. A sixth-order strain-compensated Arrhenius constitutive model was then developed to describe the coupled effects of temperature, strain rate, and strain. Full-strain-range power dissipation and flow instability maps were constructed using the dynamic material model, while optical microscopy and DEFORM-3D simulations were employed to clarify microstructural evolution and deformation inhomogeneity. The model achieved a correlation coefficient of 0.9841 and an average absolute relative error of 3.67%, demonstrating high predictive accuracy. No flow instability was detected within the investigated range, indicating excellent hot formability. The favorable compression-processing window was identified as 500–550 °C and 0.1–1 s^−1^, while the peak power-dissipation efficiency increased from 30.55% at ε = 0.2 to 32.90% at ε = 0.8. Optical-microstructural observations suggest that increasing temperature and decreasing strain rate are associated with an increasing contribution of dynamic recrystallization relative to dynamic recovery. Finite-element results further reveal pronounced spatial variations in strain, temperature, strain rate, and stress during compression. These findings provide baseline constitutive and thermomechanical information for subsequent AA3102 hot-extrusion optimization.

## 1. Introduction

AA3102 aluminum alloy, a typical Al–Mn series alloy, has become an indispensable structural material in the automotive industry due to its low density, high plasticity, and excellent corrosion resistance. It is widely used in the manufacturing of key components of flat-tube heat exchangers, such as microchannel heat exchanger tubes for automotive air-conditioning systems [1,2,3,4]. With the increasing demand for automotive lightweighting, the development of ultra-thin-walled microchannel aluminum alloy flat tubes has imposed more stringent requirements on the hot extrusion process. Therefore, a systematic investigation of the flow behavior and microstructural evolution of AA3102 aluminum alloy during high-temperature deformation is of great significance for optimizing hot processing parameters and improving the quality of formed components.

The hot workability of alloys is essentially governed by their flow behavior and microstructural evolution under various deformation conditions, including strain, strain rate, and temperature [5,6,7]. Establishing a constitutive model capable of accurately describing the coupled relationship among stress, strain, strain rate, and temperature is fundamental for predicting material deformation behavior and optimizing processing parameters [8]. In recent years, extensive studies have been conducted on the hot deformation behavior and constitutive modeling of aluminum alloys, providing a solid foundation for understanding their high-temperature rheological characteristics. Recent EBSD-based studies have further demonstrated that processing-induced changes in grain morphology, grain-boundary character, and dislocation substructure strongly influence the mechanical response of aluminum alloys [9,10]. Zhen et al. [11] performed isothermal hot compression tests on 3104 aluminum alloy using a Gleeble-1500 thermomechanical simulator at temperatures of 250–500 °C and strain rates of 0.01–10 s^−1^. After correcting the experimental data for friction and temperature effects, a modified hyperbolic-sine constitutive equation incorporating the Zener–Hollomon parameter and a seventh-order polynomial strain compensation was established, which showed good agreement with experimental results. Wang et al. [12] systematically investigated the hot deformation behavior of spray-formed 2195 Al–Cu–Li alloy. Based on the analysis of flow behavior and microstructural evolution, a strain-compensated Arrhenius constitutive model and processing maps were developed to identify the instability regions and optimal processing parameters. Xia et al. [13] studied the hot deformation behavior of 2A12-T4 aluminum alloy and performed dual corrections on the true stress–strain curves by considering temperature variation and interfacial friction. Three improved Arrhenius-type constitutive models, including strain-compensated, genetic algorithm-optimized, and K-function-modified models, were subsequently established.

Processing maps, as an effective tool for evaluating material workability and optimizing deformation parameters, have been widely applied in the study of aluminum alloys in recent years [14,15,16,17,18]. Li et al. [16] conducted hot compression tests on an Al–Cu–Mg–Ag alloy and established its constitutive equation and processing maps. Combined with EBSD characterization, it was found that the dominant dynamic softening mechanism gradually transformed from dynamic recovery (DRV) to dynamic recrystallization (DRX) with increasing temperature and decreasing strain rate. Hao et al. [19] performed isothermal compression tests on 7A21 aluminum alloy and obtained stress–strain curves within the temperature range of 350–500 °C and strain rates of 0.01–10 s^−1^. Based on the dynamic material model (DMM), processing maps at different strains were constructed, providing guidance for optimizing the hot processing parameters of the alloy.

Currently, research on the high-temperature deformation behavior and microstructural evolution of aluminum alloys mainly focuses on medium- and high-strength alloys such as the 2xxx and 7xxx series [20,21,22,23,24]. These alloys generally contain higher alloying element contents and exhibit higher flow stresses. Consequently, their deformation mechanisms and processing adaptability cannot be directly applied to low-alloyed, highly formable 3xxx-series alloys such as AA3102. In particular, the hot extrusion of ultra-thin-walled aluminum alloy flat tubes involves high-temperature large plastic deformation, yet systematic investigations on AA3102 remain limited, which restricts the optimization of its precision hot processing technology.

Systematic strain-dependent constitutive studies specifically for AA3102 remain scarce. Studies on related 3xxx alloys provide important methodological references; for example, Zhen et al. [11] developed a seventh-order strain-compensated hyperbolic-sine model for AA3104, while Fang et al. [25] compared multiple constitutive equations and established a three-dimensional processing map for AA3003. However, the fitted material parameters and processing responses of these alloys cannot be transferred directly to AA3102. Therefore, the present work focuses specifically on AA3102 and combines friction- and temperature-corrected flow data, strain-dependent Arrhenius parameters, DMM processing maps, optical-microstructural observations, and thermomechanically coupled FE simulation within the experimentally investigated compression domain.

## 2. Materials and Methods

### 2.1. Thermomechanical Simulation Tests

The investigated material was industrial as-cast AA3102 aluminum alloy supplied by A certain domestic enterprise in the form of 98 µm. Its chemical composition is shown in Table 1. To minimize compositional and microstructural variability, all compression specimens were machined from the mid-radius region at mid-height of a single ingot. Cylindrical specimens with dimensions of Φ10 mm × 15 mm were prepared. The end surfaces were sequentially ground using 400–2000 grit SiC papers to achieve Ra ≤ 0.8 μm, and the parallelism error between the two end faces was controlled within 0.02 mm.

**Figure 1:**
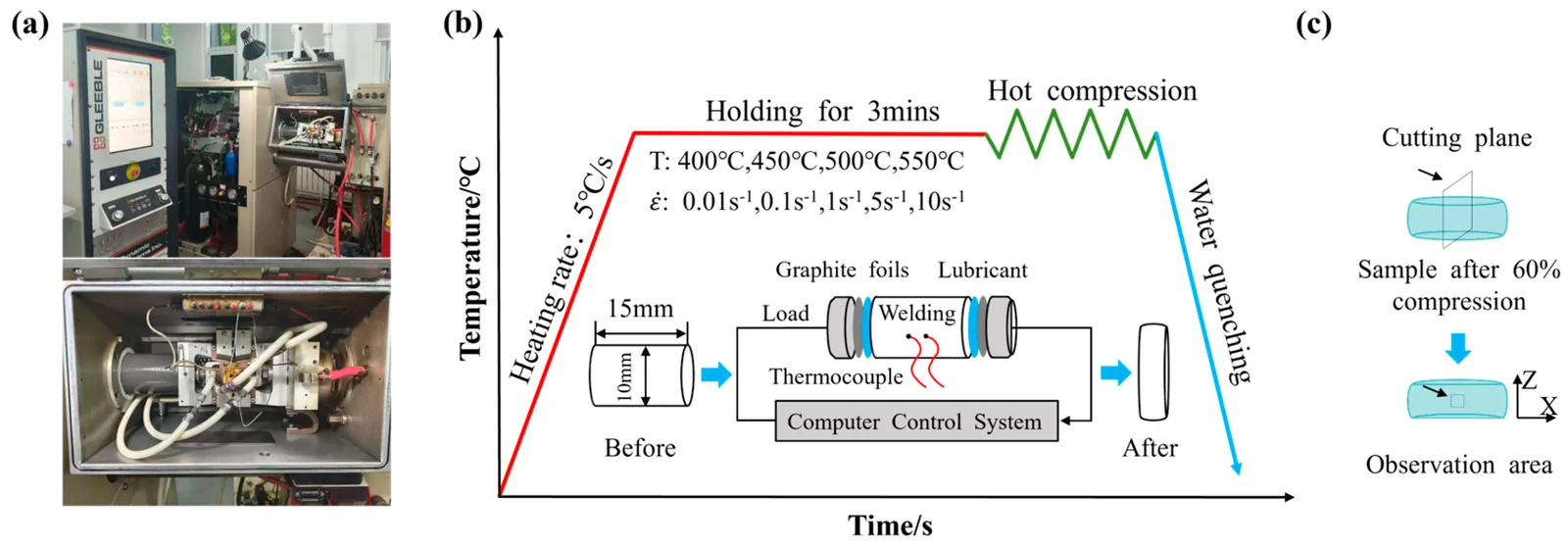
(**a**) Gleeble 3500 thermal simulation machine; (**b**) Schematic illustration of the hot compression testing procedure; (**c**) sampling method and observation plane for microstructural characterization.

The isothermal and isovelocity thermal compression test was conducted using a Gleeble-3500 thermomechanical simulator (Dynamic Systems Inc., Poestenkill, NY, USA), as shown in Figure 1a. A 0.3 mm K-type thermocouple was welded to the specimen side surface at mid-height, and the weld area was controlled within approximately 1 mm × 1 mm. A 0.2 mm high-purity graphite sheet together with high-temperature lubricant was placed at each specimen–anvil interface to reduce friction. The specimens were heated at 5 °C s^−1^ to 400, 450, 500, or 550 °C, held for 3 min, compressed at 0.01, 0.1, 1, 5, or 10 s^−1^ to a height reduction of 60%, and immediately water-quenched. Load–displacement data were continuously acquired at 100 Hz.

The thermal cycle used in the tests was as follows. The specimens were heated to the preset deformation temperatures of 400 °C, 450 °C, 500 °C, and 550 °C at a heating rate of 5 °C/s. After reaching the target temperature, the samples were held for 3 min to ensure a uniform temperature distribution and eliminate thermal gradients generated during heating. Subsequently, uniaxial hot compression was carried out at constant strain rates of 0.01 s^−1^, 0.1 s^−1^, 1 s^−1^, 5 s^−1^, and 10 s^−1^, with a total height reduction of 60% of the original height. After deformation, the specimens were immediately quenched in room-temperature deionized water with a cooling rate exceeding 100 °C/s to preserve the deformed microstructure and prevent static recrystallization or microstructural relaxation during cooling.

During the tests, the built-in data acquisition system of the simulator continuously recorded the load–displacement data with a sampling frequency of 100 Hz. The true stress–true strain curves were subsequently obtained through mathematical conversion of the recorded data. The schematic diagram of the hot compression testing procedure is shown in Figure 1b.

### 2.2. Microstructural Characterization

To investigate the deformation mechanisms under different deformation conditions and the regulatory effects of deformation process parameters on microstructural evolution, optical microscopy (OM) was employed to characterize the deformed specimens. The specimens were cut along the compression axis, and the central cross-sections were selected for grinding and polishing. The specimen sectioning scheme and observation area are shown in Figure 1c. After mechanical polishing, microstructural observations were conducted using an Axio Scope.A1 optical microscope (Carl Zeiss Microscopy GmbH, Jena, Germany).

### 2.3. Numerical Simulation

In this study, DEFORM-3D v11.0 (Scientific Forming Technologies Corporation, Columbus, OH, USA) was used to conduct numerical simulations of the hot compression process. First, the three-dimensional geometric models of the upper die, lower die, and billet were constructed using UG NX 12.0 (Siemens PLM Software, Plano, TX, USA). To reduce computational cost while maintaining simulation accuracy, the model was simplified to a half-symmetry model based on geometric symmetry and then imported into DEFORM-3D in STL format.

An adaptive meshing technique was applied to discretize the billet, with the number of mesh elements set to 10,000. Automatic remeshing was enabled to prevent numerical instability or distortion caused by severe mesh deformation during the forming process. The dies were treated as rigid bodies and therefore were not meshed; only their geometric configuration and motion boundary conditions were defined.

The material model and boundary conditions were defined as follows. A rigid–viscoplastic model was adopted in DEFORM-3D, and the parameters of the Arrhenius-type constitutive equation obtained from the corrected hot compression data were input to describe the material flow behavior. The die material was defined as AISI H13 hot-work tool steel using the built-in material database of DEFORM-3D v11.0 (Scientific Forming Technologies Corporation, Columbus, OH, USA), from which the corresponding thermophysical properties, including thermal conductivity and specific heat capacity, were obtained. The shear friction model was adopted to describe the contact behavior between the die and billet, and the friction factor was set to m=0.3  according to typical hot forging lubrication conditions.

## 3. Results and Discussion

### 3.1. Hot Deformation Behavior

**Figure 2:**
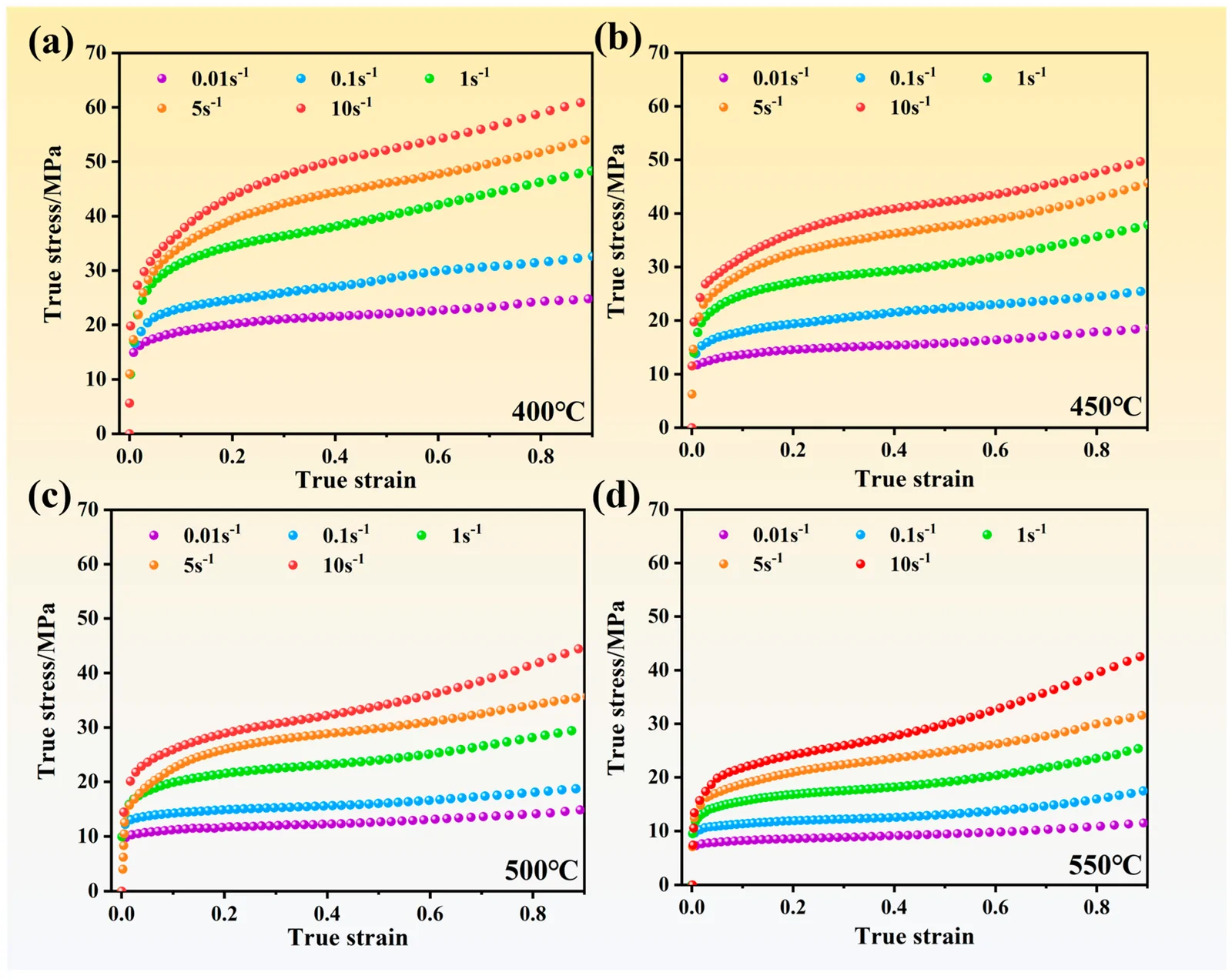
Corrected true stress–true strain curves of AA3102 aluminum alloy under different deformation conditions after friction and temperature corrections: (**a**) 400 °C, (**b**) 450 °C, (**c**) 500 °C, and (**d**) 550 °C.

During the hot compression tests, although a composite lubrication system consisting of graphite sheets and high-temperature lubricant was employed, the interfacial friction between the specimen ends and the anvils could not be completely eliminated, which results in an overestimation of the flow stress. In addition, under high strain-rate conditions, the adiabatic temperature rise generated during deformation causes the actual specimen temperature to deviate from the preset value, further resulting in deviations of the measured flow stress from the true material response. Therefore, to obtain flow data that more accurately reflect the intrinsic deformation behavior of the alloy, the original stress–strain data were corrected for both friction and temperature rise using the method proposed by Ebrahimi and Najafizadeh [26]. The corrected true stress–true strain curves are presented in Figure 2.

As shown in Figure 2, the deformation temperature and strain rate have a significant influence on the flow behavior of AA3102 aluminum alloy. In general, the flow stress increases monotonically with increasing strain rate or decreasing deformation temperature, which is consistent with the typical hot deformation characteristics of aluminum alloys.

The hot deformation process of AA3102 aluminum alloy can be divided into two stages: the work-hardening stage and the steady-state deformation stage. During the initial deformation stage, the flow stress increases rapidly with increasing strain, exhibiting a pronounced work-hardening effect. This behavior mainly originates from the massive generation, multiplication, and accumulation of dislocations, which leads to a rapid increase in dislocation density and consequently enhances the deformation resistance of the material [27,28]. As deformation proceeds, the rate of stress increase gradually decreases and eventually approaches a steady state. This phenomenon can be attributed to the activation of dynamic softening mechanisms, including DRV and DRX. With increasing dislocation density, these mechanisms are promoted, allowing dislocations to rearrange, annihilate, or move through climb and cross-slip, thereby establishing a dynamic balance between work hardening and dynamic softening.

It should be noted that under the deformation conditions investigated in this study, the work-hardening effect remains dominant, as indicated by the continuous but gradual increase in flow stress with strain, without a distinct stress peak followed by a significant decrease.

At the same deformation temperature, the stress–strain curves corresponding to different strain rates exhibit obvious differences. When the strain rate is 0.01 s^−1^, the flow stress tends to approach a steady-state regime. As the strain rate increases, the generation rate of new dislocations becomes faster, while the existing dislocations have insufficient time to move and annihilate. Consequently, the accumulated and entangled dislocations lead to a rapid increase in dislocation density and a significant rise in flow stress.

Under constant strain-rate conditions, the flow stress decreases markedly with increasing deformation temperature. This behavior is mainly attributed to the enhanced thermal activation at elevated temperatures, which reduces the critical shear stress and decreases the resistance to dislocation motion, thereby facilitating dislocation slip. For example, at a strain rate of 10 s^−1^, when the deformation temperature increases from 400 °C to 550 °C, the peak stress of the alloy decreases from 61.61 MPa to 42.92 MPa. This result directly demonstrates that increasing the deformation temperature can effectively reduce the hot deformation resistance of the alloy, providing a direct basis for optimizing hot processing parameters.

### 3.2. Constitutive Model Establishment

#### 3.2.1. Determination of Material Parameters in the Constitutive Model

During the high-temperature deformation of metallic materials, the intrinsic relationship between flow stress, deformation temperature, and strain rate is commonly described using the Arrhenius-type constitutive model based on a hyperbolic sine function, as proposed by Sellars and McTegart [29].

Under different stress levels, the constitutive equations of this model can be expressed as follows:

(1) $$\dot{\varepsilon }=A_{1}\sigma^{n_{1}}\exp\left(-Q/RT\right)$$

(2) $$\dot{\varepsilon }=A_{2\exp}{\left(\beta \sigma \right)}_{\exp}\left(-Q/RT\right)$$

(3) $$\dot{\varepsilon }=A{\left[\sin h\left(\alpha \sigma \right)\right]}^{n}\exp\left(-Q/RT\right)$$

In the above equations, A, $A_{1}$, $A_{2}$, $n_{1}$, n, α, and β are material constants; σ is the flow stress at elevated temperature (MPa); Q is the activation energy for hot deformation (J·mol^−1^); R is the universal gas constant with a value of 8.314 J·mol^−1^·K^−1^; and T is the deformation temperature (K). For the low-stress regime, Equation (1) is applicable when ασ < 0.8, whereas Equation (2) is applicable to the high-stress regime when ασ > 1.2. Equation (3) provides the unified hyperbolic-sine form over the full stress range. The stress multiplier α was determined from α = β/n_1_.

Taking the natural logarithm of both sides of Equations (1)–(3) yields:

(4) $$\ln\dot{\varepsilon }=\ln A_{1}+n_{1}\ln\sigma -Q/RT$$

(5) $$\ln\dot{\varepsilon }=\ln A_{2}+\beta \sigma -Q/RT$$

(6) $$\ln\dot{\varepsilon }=\ln A+nln[sinh(\alpha \sigma )]-Q/RT$$

**Figure 3:**
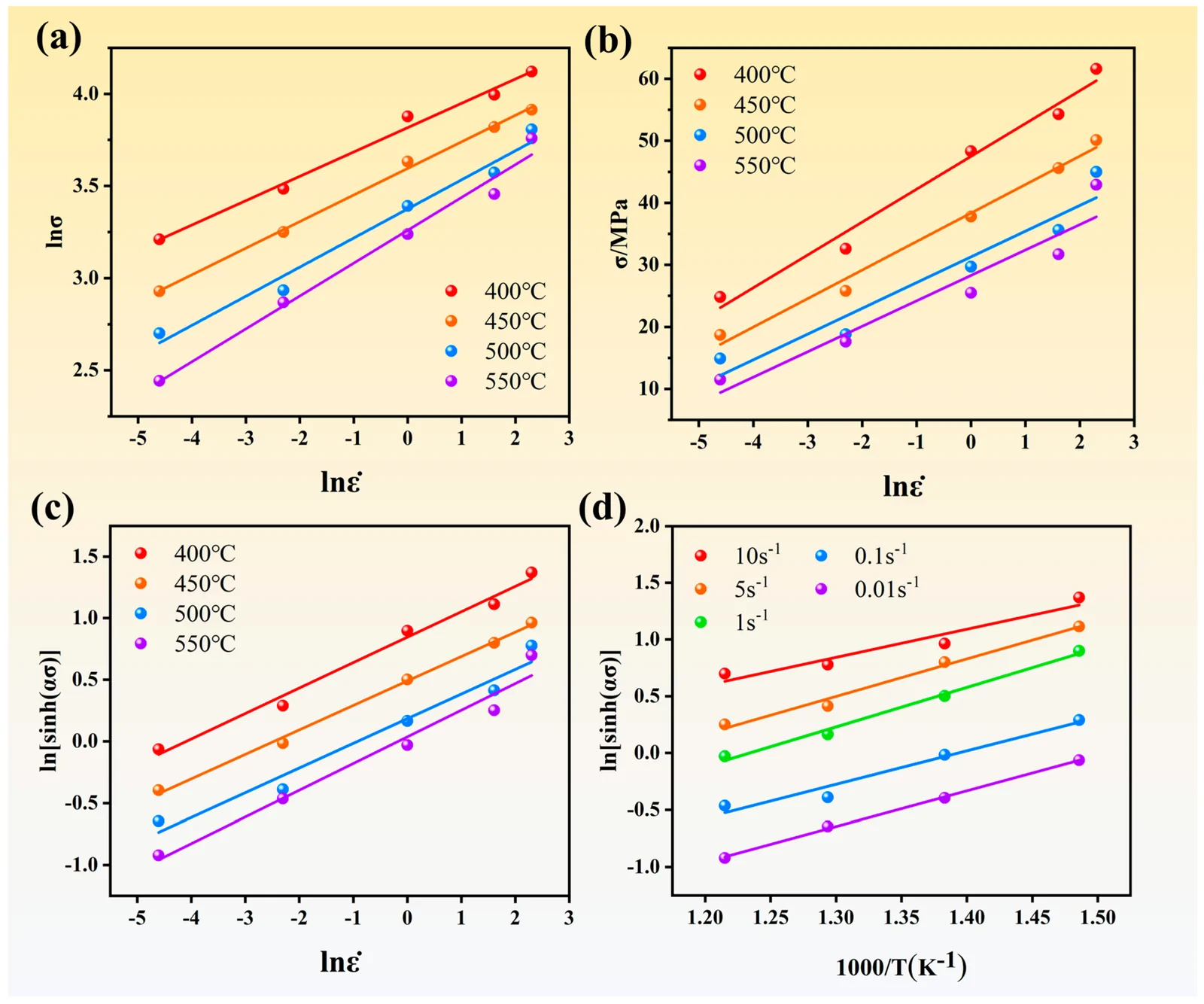
Relationship plots between (**a**) $n\sigma -\ln\dot{\varepsilon }$, (**b**) $\sigma -\ln\dot{\varepsilon }$, (**c**) $\ln[sinh(\alpha \sigma )]-\ln\dot{\varepsilon }$, and (**d**) $\ln[sinh(\alpha \sigma )]-{1000T}^{-1}.$

At a constant deformation temperature, the peak stress values obtained from the experiments were substituted into Equations (4)–(6) to establish the relationships among the parameters. Linear relationships of $ln\sigma -\ln\dot{\varepsilon }$, $\sigma -\ln\dot{\varepsilon }$, and ln[sinh(ασ)] − $\ln\dot{\varepsilon }$ were obtained, as shown in Figure 3a–c. By calculating the average slopes of these linear plots, the material constants were determined as $n_{1}$ = 6.5359, β = 0.2205, n = 4.8840, and α = 0.0337.

At each deformation temperature, ordinary least-squares linear regression was performed using the five strain-rate conditions (0.01, 0.1, 1, 5, and 10 s^−1^) for the $ln\sigma -ln\dot{\varepsilon }$, $\;\sigma -ln\dot{\varepsilon }$, and $ln[sinh(\alpha \sigma )]-ln\dot{\varepsilon }$ relationships. The slope obtained from each temperature was first determined individually, and the reported $n_{1}$, β, and n values were calculated from the mean slopes of the four temperature-specific regressions. The uncertainty of each mean slope is reported as the standard deviation of the four fitted slopes. For the activation-energy calculation, ln[sinh(ασ)] was linearly regressed against 1/T at each of the five strain rates using the four deformation temperatures (Figure 3d). The mean and standard deviation of these five slopes were then used to determine Q. Q = 193.871 kJ·mol^−1^.

Finally, the intercept of the linear fit in Figure 3c was used to determine the value of (Q/RT − ln A)/n. By substituting the values of Q, n, R, and T into the equation, the material constant A was calculated as 6.88 × 10^11^.

(7) $$Z=\dot{\varepsilon }\exp(Q/RT)=A{\left[\sin h\left(\alpha \sigma \right)\right]}^{n}$$

Based on the definition of the hyperbolic sine function, the flow stress σ can be solved from Equation (7), and Equation (8) can therefore be expressed as a function of the Zener–Hollomon parameter Z [30]:

(8) $$\sigma =\frac{1}{\alpha }\ln\left\{{\left(\frac{Z}{A}\right)}^{1/n}+{\left[{\left(\frac{Z}{A}\right)}^{1/n}+1\right]}^{1/2}\right\}$$

By substituting the previously determined values of Q, $n_{2}$, A, and α into Equation (8), the Arrhenius-type constitutive equation for AA3102 aluminum alloy can be expressed as follows:

(9) $$\sigma =29.6736\ln\left\{{\left(\frac{Z}{6.88\times 10^{11}}\right)}^{0.20475}+{\left[{\left(\frac{Z}{6.88\times 10^{11}}\right)}^{0.40950}+1\right]}^{1/2}\right\}$$

#### 3.2.2. Strain-Compensated Constitutive Equation

As indicated by Equation (9), the Arrhenius-type constitutive equation only considers the effects of deformation temperature and strain rate on the flow stress, while neglecting strain, a critical factor affecting deformation behavior. As a result, its predictive accuracy for flow stress is limited. Therefore, to more accurately predict the stress–strain relationship of metallic materials, it is necessary to develop an improved constitutive model incorporating the effect of strain.

**Figure 4:**
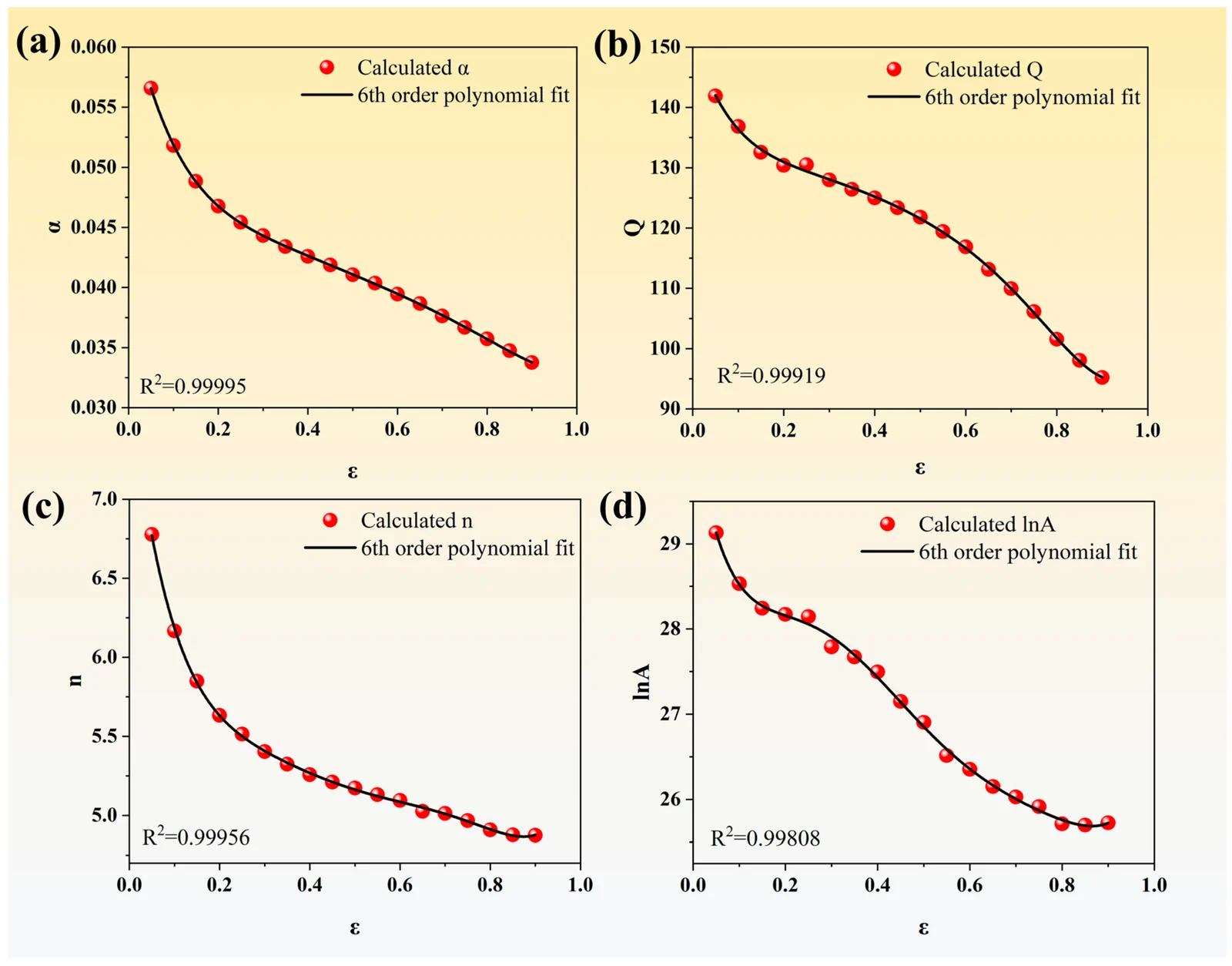
Shows the variation in material parameters in the constitutive equation with strain (**a**) α, (**b**) Q, (**c**) n, and (**d**) lnA.

It was assumed that the material parameters α, n, Q, and lnA are polynomial functions of strain ε. Within the strain range of 0.05–0.9, strain values were selected at intervals of 0.05, and the corresponding stress values were calculated. These material parameters were then determined using Equation (10). Figure 4 presents the sixth-order polynomial fitting curves of the material parameters as functions of strain. The sixth-order polynomial function calculates representative strain-dependent material parameters, as shown in Table 2. The adjusted coefficients of determination ($R^{2}$) are 0.99995, 0.99919, 0.99956, and 0.99808, respectively, indicating extremely high fitting accuracy and strong correlation. The specific coefficients of the sixth-order polynomial fitting for each material parameter are listed in Table 3.

Compared with the strain-independent Arrhenius expression in Equation (9), the strain-compensated formulation in Equation (11) explicitly accounts for the evolution of α, n, Q, and A with strain and therefore better represents the progressive change in flow response over ε = 0.05–0.90. Similar strain-compensation strategies have been reported for related 3xxx alloys, such as AA3104 [11], while multiple constitutive formulations have been compared for AA3003 [25]. The contribution of the present model is therefore not claimed to be universal superiority over these approaches, but rather an AA3102-specific calibration integrated with processing-map and FE analyses. It should also be emphasized that the same experimental matrix was used for parameter identification and error assessment; consequently, the reported R and AARE values quantify in-domain agreement rather than independent predictive validation.


(10) $$\alpha =B_{0}+B_{1}\varepsilon +B_{2}\varepsilon^{2}+B_{3}\varepsilon^{3}+B_{4}\varepsilon^{4}+B_{5}\varepsilon^{5}+B_{6}\varepsilon^{6}\;Q=C_{0}+C_{1}\varepsilon +C_{2}\varepsilon^{2}+C_{3}\varepsilon^{3}+C_{4}\varepsilon^{4}+C_{5}\varepsilon^{5}+C_{6}\varepsilon^{6}\;n=D_{0}+D_{1}\varepsilon +D_{2}\varepsilon^{2}+D_{3}\varepsilon^{3}+D_{4}\varepsilon^{4}+D_{5}\varepsilon^{5}+D_{6}\varepsilon^{6}\;\;\ln A=E_{0}+E_{1}\varepsilon +E_{2}\varepsilon^{2}+E_{3}\varepsilon^{3}+E_{4}\varepsilon^{4}+E_{5}\varepsilon^{5}+E_{6}\varepsilon^{6}$$

By incorporating the strain-dependent fitted material parameters into Equation (9), a strain-compensated constitutive model was established, as given in Equation (11).

(11) $$\sigma =\frac{1}{\alpha \left(\varepsilon \right)}\ln\left\{{\left(\frac{\dot{\varepsilon }\exp\left(Q\left(\varepsilon \right)/RT\right)}{A(\varepsilon )}\right)}^{\frac{1}{n\left(\varepsilon \right)}}+{\left[{\left(\frac{\dot{\varepsilon }\exp\left(Q\left(\varepsilon \right)/RT\right)}{A(\varepsilon )}\right)}^{\frac{2}{n\left(\varepsilon \right)}}+1\right]}^{1/2}\right\}$$

The predictive capability of the constitutive model was quantitatively assessed using the correlation coefficient (R) and the average absolute relative error (AARE), as defined in Equations (12) and (13) [31]. The corresponding expressions are given below:

(12) $$R=\frac{\sum_{i=1}^{N}\left(E_{i}-\overset{-}{E}\right)\left(P_{i}-\overset{-}{P}\right)}{\sqrt{\sum_{i=1}^{N}{\left(E_{i}-\overset{-}{E}\right)}^{2}\sum_{i=1}^{N}{\left(P_{i}-\overset{-}{P}\right)}^{2}}}$$

(13) $$AARE\left(\%\right)=\frac{1}{N}\sum_{i=1}^{N}\left|\frac{E_{i}-P_{i}}{E_{i}}\right|\times 100\%$$

In the above equations, $E_{i}$ is the experimental flow stress, $\overset{-}{E}\;$ is the average experimental flow stress, $P_{i}\;$ is the predicted flow stress, $\overset{-}{P}\;$ is the average predicted flow stress, and N is the total number of experimental data points.

**Figure 5:**
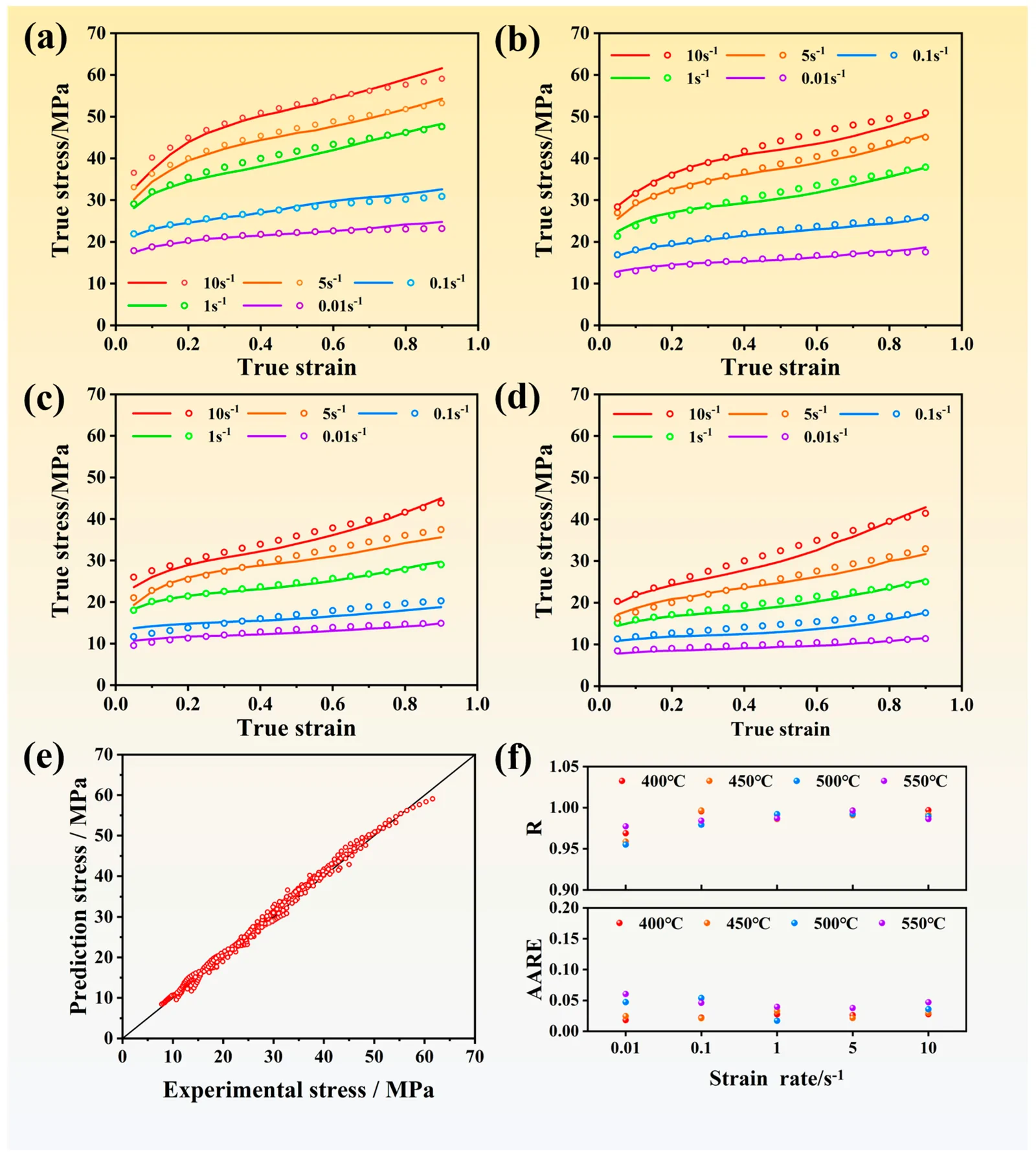
Comparison between the predicted flow stresses (symbols) and experimental flow stresses (solid lines) of AA3102 aluminum alloy: (**a**) 400 °C, (**b**) 450 °C, (**c**) 500 °C, (**d**) 550 °C, (**e**) comparison between predicted and experimental stress values, and (**f**) values of R and AARE.

As can be seen from Figure 5a–e, the agreement between the predicted values and the experimental values is quite high. Under different deformation conditions, the R value between the test values and the predicted values is relatively high, while the AARE value is relatively low, as shown in Figure 5f. The correlation coefficient R of the model’s predicted values and the test values is 0.9841. The R values at each temperature are: 0.9779, 0.9874, 0.9923, and 0.9788. The average relative error AARE is 3.67%, and the AARE values at each temperature are: 4.21%, 3.42%, 2.76%, and 4.29%. These results indicate that the developed strain-compensated Arrhenius constitutive model can relatively accurately describe the intrinsic relationship among flow stress, strain, strain rate, and deformation temperature during the hot deformation of AA3102 aluminum alloy. It should be emphasized that the same experimental matrix was used for parameter identification and error assessment. Therefore, the reported R and AARE values quantify the agreement within the calibrated deformation domain rather than independent predictive validation. The sixth-order polynomial is used here as an empirical strain-compensation function to represent the nonlinear evolution of the material parameters with strain. Independent deformation conditions are required to establish extrapolative capability outside the present dataset.

### 3.3. Processing Maps of AA3102 Aluminum Alloy

Processing maps are important tools established on the dynamic materials model (DMM) and are widely used to analyze the hot deformation behavior of metallic materials and optimize hot processing parameters. The model was proposed by Prasad et al. [32] based on the principles of irreversible thermodynamics and continuum mechanics. Its core concept is to treat the hot deformation process of metallic materials as an energy dissipation system, in which the total power dissipation, P, can be divided into two complementary parts, namely, the content dissipated through plastic deformation, G, and the co-content dissipated through microstructural evolution, J. Specifically, G represents the energy consumed during plastic deformation, most of which is converted into heat, while only a small portion is stored in the material in the form of defects. In contrast, J corresponds to the energy dissipated through microstructural evolution processes during deformation, such as DRV, DRX, and phase transformation. The total power dissipation, P, can be expressed as follows:

(14) $$P=\sigma \cdot \dot{\varepsilon }=G+J=\int_{0}^{\dot{\varepsilon }}\sigma d\dot{\varepsilon }+\int_{0}^{\sigma }\dot{\varepsilon }d\sigma$$

At a given strain and deformation temperature, the flow stress of the metallic material can be expressed as:

(15) $$\sigma =K{\dot{\varepsilon }}^{m}$$

where K is a material constant and m is the strain-rate sensitivity exponent. At a constant strain and deformation temperature, m can be expressed as [33]:

(16) $$m={\left(\frac{\partial \ln\sigma }{\partial \ln\dot{\varepsilon }}\right)}_{\varepsilon ,T}$$

Substituting Equation (15) into Equation (14) yields:

(17) $$P=G+J=\int_{0}^{\dot{\varepsilon }}K{\dot{\varepsilon }}^{m}d\dot{\varepsilon }+\int_{0}^{\sigma }{\left(\sigma /K\right)}^{1/m}d\sigma =\frac{\sigma \dot{\varepsilon }}{m+1}=\frac{\sigma \dot{\varepsilon }m}{m+1}$$

According to Equation (17), the strain-rate sensitivity exponent m determines the partitioning relationship between the dissipator content G and the co-content J. Under the ideal linear dissipation condition, m=1, at which point the co-content J reaches its maximum value of $(\sigma \dot{\varepsilon })/2$. For a nonlinear dissipation process, the power dissipation efficiency can be characterized by a dimensionless parameter, η, which is expressed as follows:

(18) $$\eta =\frac{J}{J_{\max}}=\frac{2m}{m+1}\;\;\;\;\;\;\;$$

The parameter η reflects the proportion of the total dissipated energy that is consumed by microstructural evolution during the hot deformation of metallic materials. In general, a higher η value indicates that a larger fraction of the dissipated energy is associated with microstructural evolution processes, such as DRV and DRX, and thus suggests better hot workability under the corresponding deformation conditions. However, a high power dissipation efficiency is not, by itself, a sufficient indication of good hot workability. In some cases, abnormal microstructural changes caused by plastic flow instability tend to also result in an anomalously high power dissipation efficiency. Therefore, it is necessary to identify the plastic instability regions within the deformation domain using an instability criterion. Based on the principle of maximum entropy production rate proposed by Ziegler, Prasad et al. [34] established the following flow instability criterion:

(19) $$\xi \left(\dot{\varepsilon }\right)=\frac{\partial \ln\left[m/\left(m+1\right)\right]}{\partial \ln\dot{\varepsilon }}+m$$

It is generally accepted that when $\xi (\dot{\varepsilon })<0$, the material is in a flow instability state, and the larger the value of $|\xi (\dot{\varepsilon })|$, the more severe the flow instability.

**Figure 6:**
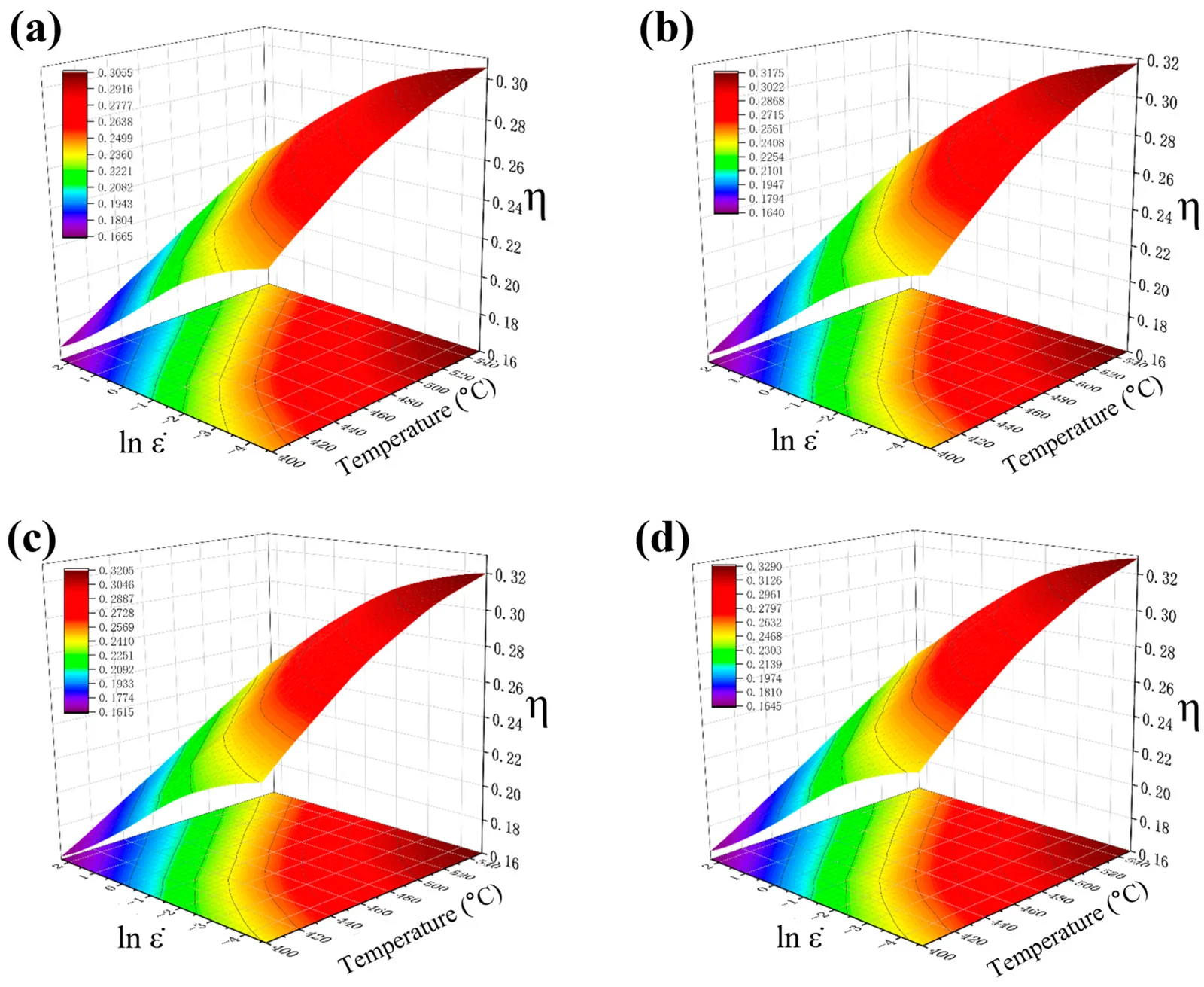
Power dissipation efficiency maps of AA3102 aluminum alloy at different true strains: (**a**) 0.2, (**b**) 0.4, (**c**) 0.6, and (**d**) 0.8.

According to Equation (18), the power dissipation efficiency maps of AA3102 aluminum alloy were calculated and constructed within the deformation temperature range of 400–550 °C, the strain-rate range of 0.01–10 s^−1^, and the true strain range of 0.2–0.8, as shown in Figure 6. It can be seen from Figure 6 that the processing maps are significantly affected by deformation temperature, strain rate, and true strain. When the deformation temperature is within 400–450 °C, and the strain rate is higher than 1 s^−1^, the η values corresponding to all true strains (0.2–0.8) remain below 0.20, indicating that the material is in a low power dissipation region. In this region, only a limited proportion of the deformation energy is consumed by microstructural evolution, while the dislocation multiplication rate exceeds the dynamic softening rate, leading to dislocation accumulation and an increase in internal stress. As a result, localized adiabatic shear phenomena may occur, which is unfavorable for the stable progression of hot working.

When the deformation temperature increases above 450 °C and the strain rate decreases below 1 s^−1^, the η value shows a gradual upward trend. Once η exceeds 0.25, the material enters a high-efficiency and stable power dissipation region, in which the deformation energy can continuously promote microstructural evolution and thereby facilitate stable and homogeneous plastic deformation. In addition, the peak power dissipation efficiency increases with increasing true strain, rising from 0.3055 at a true strain of 0.2 to 0.3290 at a true strain of 0.8, corresponding to an increase of 7.7%. This variation can be attributed to the continuous accumulation of stored energy with increasing true strain. Meanwhile, part of the stored energy is consumed by dislocation rearrangement and annihilation during DRV as well as by DRX. Under higher true strain conditions, the elevated temperature further promotes DRV and DRX, leading to more pronounced microstructural evolution and, consequently, higher power dissipation efficiency. Therefore, in practical hot working, the high-efficiency and stable power dissipation region should be preferentially selected as the processing window.

**Figure 7:**
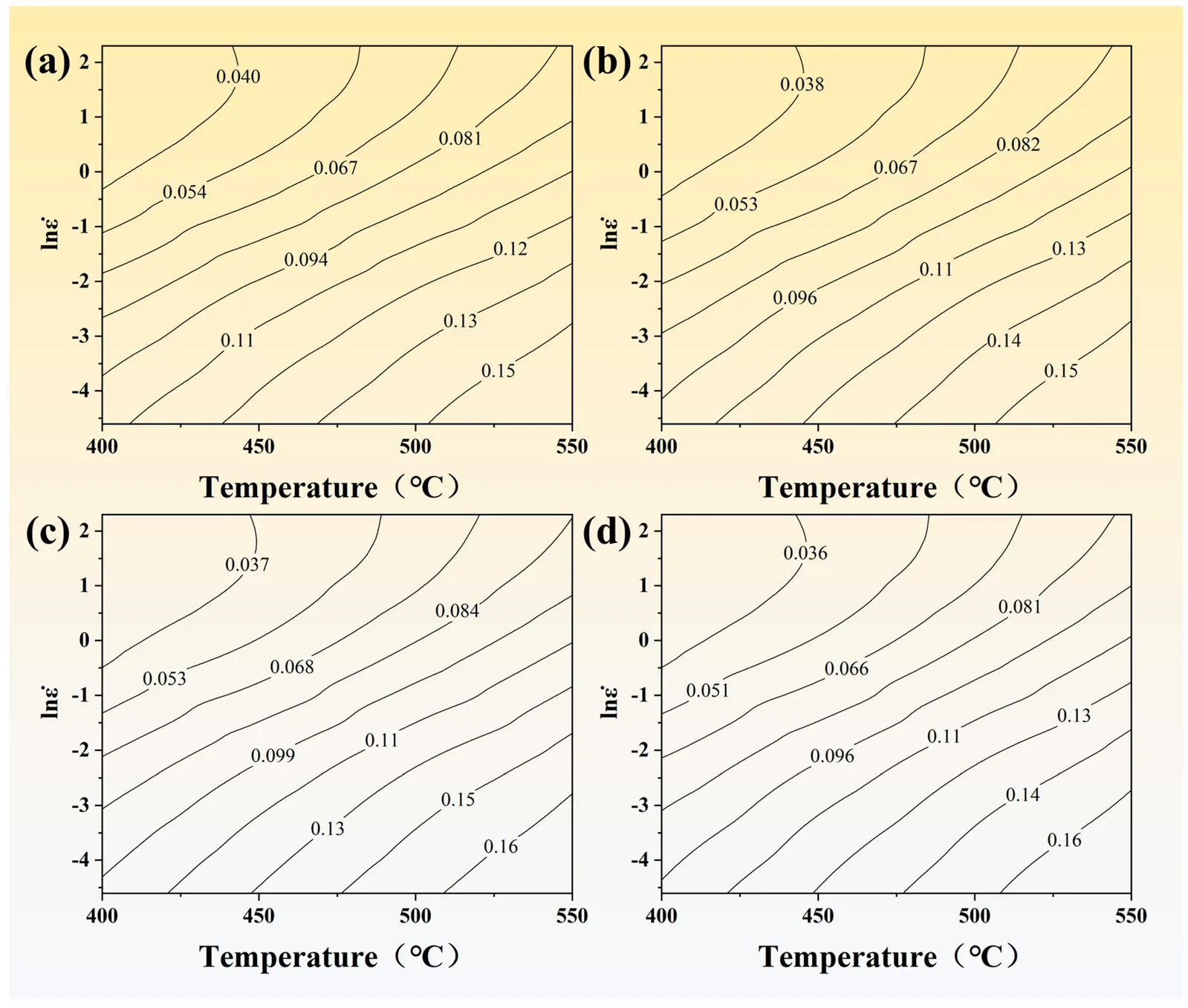
Flow instability maps of AA3102 aluminum alloy at different true strains: (**a**) 0.2, (**b**) 0.4, (**c**) 0.6, and (**d**) 0.8.

According to Equation (19), the flow instability parameter ξ of AA3102 aluminum alloy was calculated within the deformation temperature range of 400–550 °C, the strain-rate range of 0.01–10 s^−1^, and the true strain range of 0.2–0.8, and the corresponding flow instability maps were constructed, as shown in Figure 7. As can be seen from Figure 7, although the ξ values exhibit some variations with true strain, they remain greater than zero throughout the entire experimental parameter range, and no region with ξ<0 is observed. This indicates that no flow instability occurs in AA3102 aluminum alloy under the deformation conditions investigated in this study, namely deformation temperatures of 400–550 °C and strain rates of 0.01–10 s^−1^. The absence of ξ < 0 indicates that the DMM criterion does not predict a macroscopic flow-instability domain within the tested compression range. This result does not imply complete microstructural uniformity. Conditions with low power-dissipation efficiency may still produce heterogeneous deformation morphologies, mixed grains, or local grain coarsening without satisfying the mathematical instability criterion ξ < 0. Therefore, the processing map and the optical microstructures should be interpreted as complementary indicators of macroscopic flow stability and microstructural quality, respectively.

According to hot working theory, when the material is in a stable domain (ξ>0), a higher power dissipation efficiency η generally corresponds to better hot workability. Combining the analysis of the power dissipation efficiency maps presented above, the optimal hot deformation processing window for AA3102 aluminum alloy can be identified as a deformation temperature of 500–550 °C and a strain rate of 0.1–1 s^−1^.

### 3.4. Synergistic Effects of Strain Rate and Temperature on Microstructural Evolution

**Figure 8:**
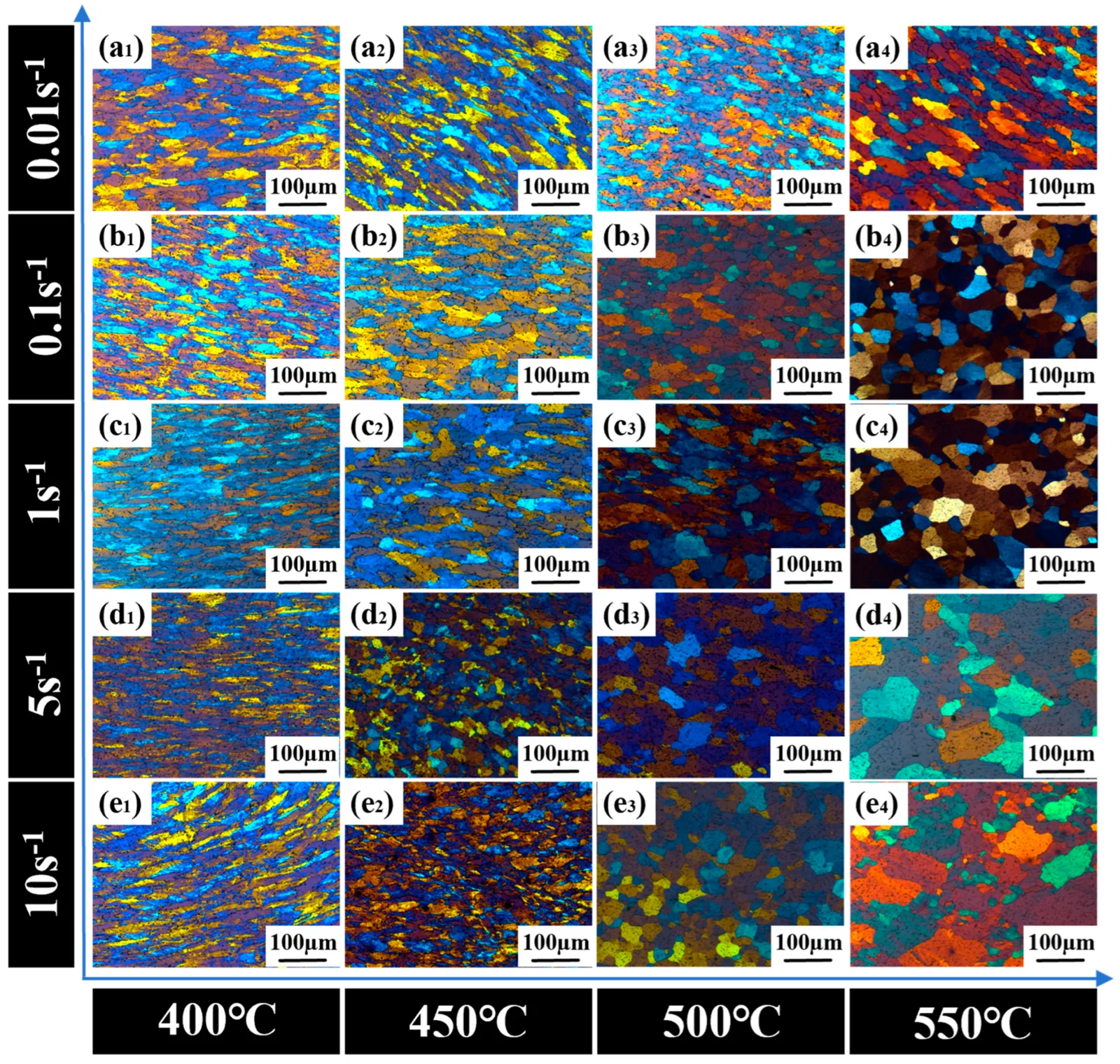
Optical microstructures of AA3102 aluminum alloy under different hot-deformation conditions. Rows (**a**–**e**) correspond to strain rates of 0.01, 0.1, 1, 5, and 10 s^−1^, respectively; columns (**1**–**4**) correspond to deformation temperatures of 400, 450, 500, and 550 °C, respectively.

Figure 8 shows the optical microstructures of AA3102 aluminum alloy under different hot deformation conditions. Comparable grain refinement and microstructural homogenization have also been achieved in semisolid aluminum alloys through severe plastic deformation [35]. In the low-temperature range of 400–450 °C, the microstructures are predominantly characterized by elongated or fibrous deformed grains, which is consistent with a strong dynamic-recovery contribution and limited recrystallization. As the temperature increases to 500–550 °C, particularly at 0.1–1 s^−1^, the microstructure progressively evolves toward more equiaxed grain morphologies, suggesting an increased contribution of dynamic recrystallization. However, optical microscopy alone cannot quantitatively distinguish recovered, substructured, and recrystallized regions. Therefore, the DRV/DRX interpretation in the present work should be regarded as a morphology-based inference supported by the flow response, processing-map trends, and previous studies rather than direct crystallographic identification. As an Al–Mn alloy, AA3102 has relatively high stacking-fault energy, which favors dislocation rearrangement through cross-slip and climb and is consistent with a strong dynamic-recovery contribution. In addition, Mn/Fe-containing dispersoids or intermetallic particles commonly reported in Al–Mn alloys may retard subgrain- and grain-boundary migration through a Zener-type pinning effect [36,37]. Because direct particle/phase characterization was not performed in the present work, this mechanism is discussed only as a plausible literature-supported contribution rather than as direct phase identification. In particular, at relatively high strain rates (5–10 s^−1^), the short deformation time and limited thermal activation result in microstructures still dominated by fibrous deformed grains, with very limited recrystallization nucleation.

As the deformation temperature increases to 500–550 °C, the dependence of microstructural evolution on strain rate becomes more pronounced. Under low strain-rate conditions of 0.1–1 s^−1^ (Figure 8(b3–c4)), atomic diffusion becomes more sufficient, and the DRX process is significantly promoted. Dynamic recrystallization under the combined effects of elevated temperature, intense plastic deformation, and high local strain rate has also been reported in processed aluminum alloys [38]. As a result, the grain size gradually increases, and relatively uniform equiaxed recrystallized grains are formed. Under these conditions, the material exhibits relatively high power dissipation efficiency, and the hot deformation energy can be effectively converted into the driving force for DRX, leading to improved microstructural uniformity. In contrast, at high strain rates of 5–10 s−1, the short deformation time and pronounced adiabatic heating limit the development of a homogeneous softened microstructure. Coarse equiaxed regions may coexist with unrecovered deformation bands, leading to pronounced microstructural heterogeneity. These features are interpreted as indicators of suboptimal microstructural evolution rather than direct evidence of DMM flow instability.

Based on the microstructural observations and processing map analysis, AA3102 aluminum alloy exhibits the optimal hot workability within the range of 500–550 °C and 0.1–1 s^−1^. Under these conditions, the microstructure is characterized by uniform equiaxed recrystallized grains, corresponding to relatively high power dissipation efficiency and no apparent risk of flow instability, thereby enabling stable hot plastic forming. In contrast, under low-temperature and high-strain-rate conditions (400–450 °C, 5–10 s^−1^), insufficient DRV leads to the formation of fibrous microstructures and high dislocation density, which are likely to cause plastic anisotropy and forming cracks. Meanwhile, under high-temperature and high-strain-rate conditions (500–550 °C, 5–10 s^−1^), mixed grain structures and local grain coarsening are prone to occur, both of which are detrimental to the uniformity and stability of the mechanical properties.

### 3.5. Deform Simulation of Hot Compression

The constitutive equation of AA3102 aluminum alloy obtained above [Equation (11)] was imported into the finite element simulation software DEFORM-3D to establish the material database of AA3102 aluminum alloy, which can be used for the simulation and analysis of its compressive plastic deformation behavior [39]. In the present study, the deformation behavior of AA3102 aluminum alloy under hot compression was mainly simulated, with particular attention paid to the distributions and evolution of strain, strain rate, temperature, and stress when the specimen was compressed to a true strain of 0.9 at a deformation temperature of 500 °C and a strain rate of 1 s^−1^. The simulated specimen had the same dimensions as the experimental sample (ϕ10 mm × 15 mm), and one-half of the cylindrical specimen was selected for numerical calculation. The present FE model represents upsetting of the cylindrical compression specimen and therefore does not reproduce the complex material flow, die geometry, frictional contact, and heat-transfer conditions of microchannel-tube extrusion. Geometry-specific extrusion simulation and validation are required before direct process transfer.

**Figure 9:**
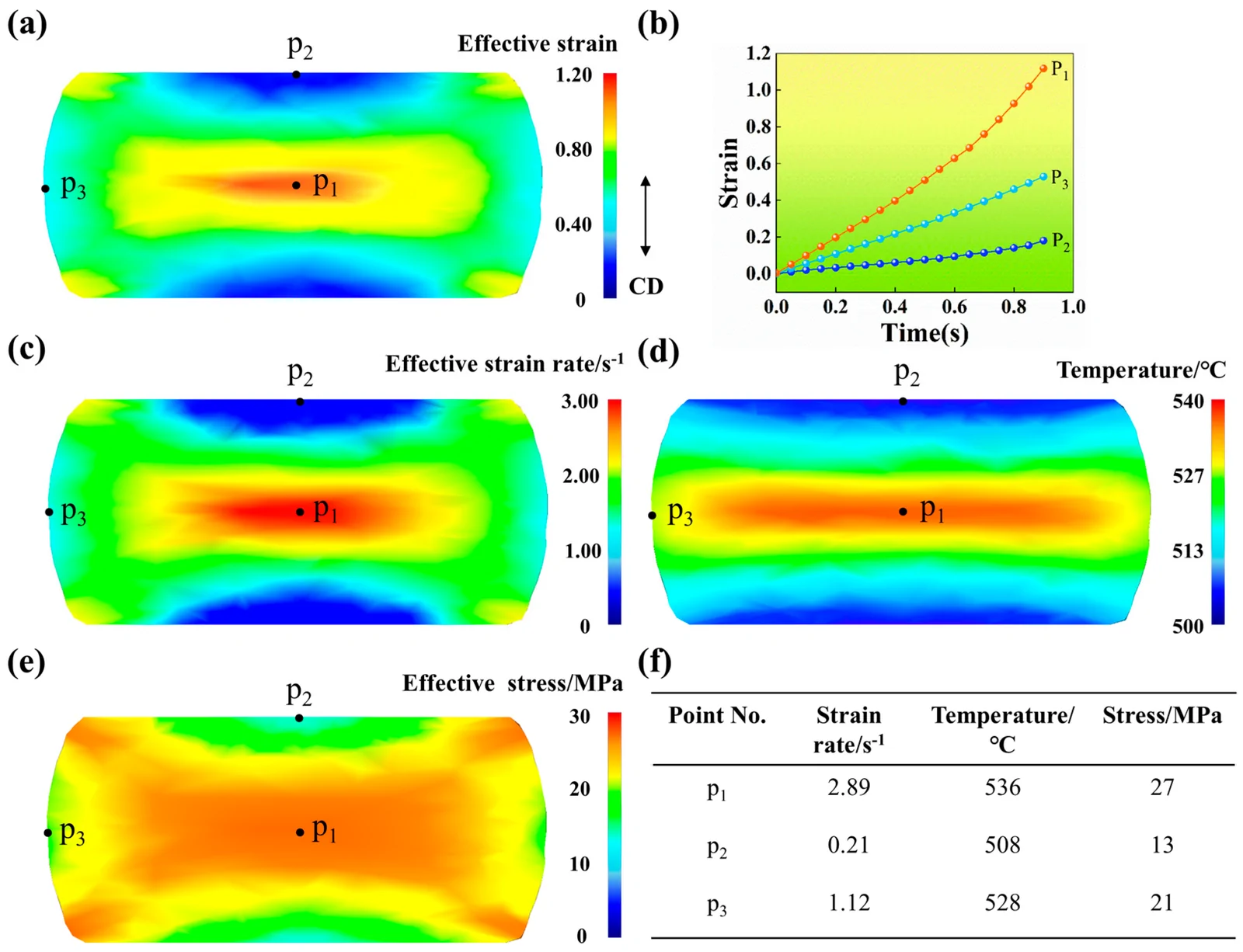
Finite element simulation of specimen under hot compression to true strain of 0.9 at 500 °C and 1 s^−1^. (**a**) Contour of effective strain, (**b**) Strain-time curves at characteristic points ($P_{1}$), ($P_{2}$) and ($P_{3}$), (**c**) Contour of effective strain rate, (**d**) Contour of temperature distribution, (**e**) Contour of effective stress, and (**f**) Simulated quantitative parameters of strain rate, temperature and effective stress at ($P_{1}$), ($P_{2}$) and ($P_{3}$).

Figure 9 presents the thermos-mechanical coupled finite element simulation results of the compression process of AA3102 aluminum alloy, where the vertical direction represents the compression direction (CD). The equivalent strain contour in Figure 9a shows that the strain distribution within the specimen is markedly non-uniform, which is caused by inhomogeneous deformation induced by friction between the specimen ends and the anvils. Among the selected positions, the central region (point $P_{1}$) exhibits the maximum strain of 1.12 and can therefore be regarded as the easy-deformation zone. In contrast, the central position of the end face in contact with the anvils (point $P_{2}$) shows the minimum strain of only 0.18 and can thus be considered the difficult-deformation zone. Owing to the barreling effect, the strain state at the cylindrical surface (point $P_{3}$) is relatively complex, with a strain value of 0.53. The strain–time curves in Figure 9b further illustrate the deformation evolution at these three representative positions. The onset of DRX is generally sensitive to the local strain level. The FE results show that $P_{2}$ experiences a much lower accumulated strain than $P_{1}$, suggesting that the local thermomechanical conditions at $P_{2}$ are less favorable for extensive recrystallization, whereas the higher strain and temperature at $P_{1}$ are more favorable for dynamic softening and possible recrystallization. Because the FE model does not directly resolve microstructural state variables and no EBSD analysis was performed, these statements should be interpreted as thermomechanical tendencies rather than direct confirmation of the local DRX fraction.

The onset of DRX is generally sensitive to the local strain level. The FE results show that P2 experiences a much lower accumulated strain than P1, suggesting that the local thermomechanical conditions at P2 are less favorable for extensive recrystallization, whereas the higher strain and temperature at P1 are more favorable for dynamic softening and possible recrystallization. Because the FE model does not directly resolve microstructural state variables and no EBSD analysis was performed, these statements should be interpreted as thermomechanical tendencies rather than direct confirmation of the local DRX fraction.

The non-uniform deformation of the compressed specimen leads to non-uniform distributions of strain rate, stress, and temperature within the specimen, as shown in Figure 9c–f. In this study, three representative points, $P_{1}$, $P_{2}$, and $P_{3}$, were selected for quantitative analysis. The results show that the equivalent strain rate, temperature, and equivalent stress all exhibit significant spatial heterogeneity. Among them, the values of all these physical quantities at the specimen center ($P_{1}$) are the highest, whereas those at the edge region ($P_{2}$) are the lowest. The detailed simulation data for each point are listed in the table shown in Figure 9f.

It is worth noting that although the nominal strain rate during compression was set to 1 s^−1^, the actual strain rates at different locations within the specimen differ significantly when the true strain reaches 0.9, as shown in Figure 9c. Specifically, the strain rate at $P_{2}$ is 0.21 s^−1^, whereas that at $P_{1}$ reaches 2.89 s^−1^, which is nearly three times the initial preset value. This result is fully consistent with the rapid strain accumulation observed in the central region. Meanwhile, the actual temperature at $P_{1}$ reaches 536 °C (Figure 9d), which is 36 °C higher than the preset deformation temperature. This temperature rise originates from the conversion of mechanical work into heat during plastic deformation. Since the deformation in the central region is more severe, the heat generation effect is more pronounced, resulting in the largest temperature increase. It can therefore be inferred that the central region of the specimen is subjected to deformation conditions of both high temperature and high strain rate, and the recrystallized grain size in this region cannot be strictly estimated based solely on the initial deformation parameters. On this basis, to achieve a more reasonable control of recrystallized grain size, slightly lower deformation temperatures and strain rates may be considered in the compression process, provided that deformation compatibility and continuity can still be maintained.

From the stress distribution shown in Figure 9e, the stress in some local regions of the specimen exceeds the steady-state stress value of 28 MPa at a true strain of 0.9, as indicated in Figure 2c. However, the stress values at the three representative points $P_{1}$*–*$P_{3}$ are all lower than this steady-state stress. This phenomenon can be reasonably interpreted by combining the strain-rate and temperature distributions with the stress–strain curves shown in Figure 2. Taking point $P_{1}$ as an example, its actual strain rate is higher than the preset value, which tends to increase the steady-state stress, whereas its actual temperature is also higher than the preset value, which tends to decrease the steady-state stress. Overall, the effect of temperature on the steady-state stress is more significant than that of strain rate, ultimately leading to an actual stress at $P_{1}$ lower than the steady-state stress. In addition, other influencing factors may also contribute to this phenomenon, which still requires further investigation and verification in future work. The present FE model represents upsetting of the cylindrical compression specimen and does not reproduce the complex material-flow path, die geometry, interfacial friction, and heat-transfer conditions of microchannel-tube extrusion. Geometry-specific extrusion simulation and experimental validation are therefore required before direct process transfer.

## 4. Conclusions

This work systematically investigates the hot deformation behavior, microstructural evolution, and inhomogeneous forming characteristics of AA3102 aluminum alloy via hot compression tests, constitutive modeling, processing map analysis, and finite element simulation. The main conclusions are summarized as follows:

(1) A sixth-order strain-compensated Arrhenius formulation was established for AA3102 over the investigated deformation domain. The model shows good agreement with the experimental dataset, with R = 0.9841 and a pooled AARE of 3.67%. Because the same deformation matrix was used for parameter identification and error assessment, these statistics represent in-domain fitting performance rather than independent predictive validation. The strain-dependent formulation captures the evolution of the material parameters over ε = 0.05–0.90, while further validation under unseen deformation conditions remains necessary.
(2) The DMM analysis identified a favorable uniaxial-compression window of 500–550 °C and 0.1–1 s^−1^. The peak power-dissipation efficiency increased from 0.3055 at ε = 0.2 to 0.3290 at ε = 0.8, and no ξ < 0 instability domain was obtained within the tested range. This window is a process reference for subsequent extrusion optimization rather than a directly validated extrusion window.
(3) The flow response and optical microstructures indicate a strong DRV contribution at 400–450 °C and an increasing DRX contribution with increasing temperature and decreasing strain rate. FE analysis further demonstrates pronounced local thermomechanical heterogeneity during compression.

## Figures and Tables

**Table 1:** Chemical composition of AA3102 Alloys (%).

Mn	Fe	Cu	Si	Mg	Cr	Ti	Al
0.40	0.56	0.09	0.38	0.03	0.04	0.03	Bal

**Table 2:** Representative strain-dependent material parameters calculated from the sixth-order polynomial functions.

ε	α	n	Q	lnA
0.05	0.05649	6.7701	142.000	29.1312
0.20	0.4672	5.6316	130.947	28.1572
0.40	0.4259	5.2682	125.197	27.4310
0.60	0.03944	5.0854	116.640	26.3573
0.80	0.3569	4.9121	101.759	25.7586
0.90	0.03377	4.8769	95.249	25.7222

**Table 3:** Fitting coefficients of the 6th-order polynomial for α, n, Q and lnA.

i	α	Q	n	lnA
	$B_{i}$	$C_{i}$	$D_{i}$	$E_{i}$
0	0.0636	7.7397	151.4346	30.4231
1	−0.1732	−24.4813	−240.4060	−35.6110
2	0.6963	116.3669	1193.2779	229.9907
3	−1.6394	−314.2709	−3440.6331	−748.6451
4	2.1790	474.7249	5489.9049	1207.1476
5	−1.5495	−373.3277	−4692.6998	−951.4881
6	0.4563	118.5380	1638.7741	294.7712

## Data Availability

The original contributions presented in this study are included in the article. Further inquiries can be directed to the corresponding authors.
